# Protocol for the health economic evaluation of nature-based social prescribing against loneliness alongside the RECETAS trials

**DOI:** 10.1186/s12889-026-27698-2

**Published:** 2026-05-09

**Authors:** Veronika Papon, Sibylle Puntscher, Marjan Arvandi, Beate Jahn, Jill S. Litt, Laura Coll-Planas, Montse Masó-Aguado, Kaisu H. Pitkala, Marja-Liisa Laakkonen, Hanna-Maria Roitto, Iva Holmerová, Alzbeta Bartova, Mat Jones, Michael Drummond, Uwe Siebert, Ursula Rochau

**Affiliations:** 1 Institute of Public Health, Medical Decision Making and Health Technology Assessment, Department of Public Health, Health Services Research and Health Technology Assessment, UMIT TIROL - University for Health Sciences and Technology, Hall in Tirol, Austria; 2 Institute for Global Health (ISGlobal), Barcelona Biomedical Research Park (PRBB), Doctor Aiguader, 88, Barcelona, 08003 Spain; 3 CIBER Epidemiología y Salud Pública (CIBERESP), Madrid, Spain; 4 Universitat Pompeu Fabra (UPF), Barcelona, Spain; 5 Research Group On Methodology, Methods, Models and Outcomes of Health and Social Sciences (M3O), Faculty of Health Sciences and Welfare, Centre for Health and Social Care Research (CESS), University of Vic-Central University of Catalonia (UVic-UCC), Carrer de La Sagrada Família, 7, Barcelona, 08500 Vic Spain; 6 Institute for Research and Innovation in Life Sciences and Health in Central Catalonia (IRIS-CC), Vic, Spain; 7 University of Helsinki, Department of General Practice and Helsinki University Hospital, Unit of Primary Health Care, Helsinki, Finland; 8 City of Helsinki, Department of Social Services and Health Care, Helsinki, Finland; 9 Centre of Expertise in Longevity and Long-Term Care, Faculty of Humanities, Charles University, Prague, Czech Republic; 10 Centre for Public Health and Wellbeing, University of the West of England, Bristol, UK; 11 Centre for Health Economics, University of York, York, UK; 12 Center for Health Decision Science, Departments of Epidemiology and Health Policy & Management, Harvard Chan School of Public Health, Boston, MA USA; 13 Institute for Technology Assessment, Department of Radiology, Massachusetts General Hospital, Harvard Medical School, Boston, MA USA

**Keywords:** Cost-effectiveness analysis alongside the trial, Study protocol, Health-economic evaluation, Loneliness, Nature-based social prescribing, RECETAS, Health-related quality of life, Capabilities

## Abstract

**Background:**

Loneliness has become a global public health problem associated with serious diseases and early mortality. A promising non-medical group-based strategy to address loneliness is nature-based social prescribing (NBSP). The aim of this paper is to describe the design of a health economic evaluation of NBSP interventions alongside trials conducted in the EU H2020 project RECETAS.

**Methods:**

The health economic evaluation will be conducted alongside the RECETAS randomized controlled trials in Barcelona, Prague, and Helsinki, and comprises cost-utility, cost-effectiveness, and cost-capability analyses. Depending on each study site’s specific setting and target population, the economic evaluation will be performed from a societal and/or a healthcare perspective. NBSP interventions, which include referring lonely people to participate in activities organized in a natural environment and providing a selection of nearby nature-based community resources, will be evaluated against usual care. Effectiveness measures include health-related quality of life assessed with the EQ-5D-5 L questionnaire, changes in loneliness obtained from the De Jong Gierveld Loneliness Scale, and capability derived from the ICECAP-A questionnaire. Direct healthcare costs, indirect and informal costs as well as the actual costs of the intervention will be included. The data on health outcome and health care resource utilization will be collected at baseline and at three-, six- and twelve-months follow-up of the trial. The incremental cost-utility ratio, incremental cost-effectiveness ratio, and incremental cost-capability ratio will be calculated. National willingness-to-pay thresholds will be applied where available.

**Discussion:**

Our study protocol provides a systematic framework designed prospectively for conducting the health economic evaluation of the NBSP intervention for people suffering from loneliness. Through the publication of the study protocol, the research process becomes transparent and reproducible. This reduces bias when interpreting the results and conclusions and ensures good evidence-based practice, so that decision makers can provide evidence-based recommendations without arbitrariness to support NBSP implementation.

**Trial registration:**

Barcelona (Spain) trial ClinicalTrials.gov, ID NCT05488496. Registered 29 July 2022 Prague (Czech Republic) trial ClinicalTrials.gov, ID NCT05522140. Registered August 25, 2022 Helsinki (Finland) trial ClinicalTrials.gov, ID NCT05507684. Registered August 12, 2022.

## Background

Loneliness has emerged as a significant public health concern, as highlighted by the United States Surgeon General Dr. Vivek Murthy in 2023, who referred to this issue as an epidemic [1]. A recent report from the World Health Organization reinforces this assessment, confirming that loneliness is a growing public health issue on a global scale with considerable social and economic expenses [2].

Loneliness is defined as “the unpleasant experience that occurs when a person’s network of social relations is deficient in some important way, either quantitatively or qualitatively” [3]. Despite the fact that maintaining social contact has never been easier due to technology, the prevalence of loneliness remains high around the world [4, 5]. Loneliness transcends geographical borders, affects all generations, and is related to serious diseases such as a cognitive decline or dementia [6], diabetes [7], depression and anxiety or cardiovascular diseases [8, 9] and can lead to early mortality [10–12].

Traditionally, social support has been identified as an important determinant in alleviating loneliness [13]. One upcoming idea for addressing loneliness as a global public health problem is social prescribing (SP), defined as a healthcare approach that connects individuals to non-medical community resources and activities to improve their overall well-being [14]. As a subtype of SP, nature-based social prescribing (NBSP), which includes green and blue social prescribing, focuses on individual and/or group-based actions related to nature, such as gardening, fishing, bird watching or hiking [15, 16].

While evidence on the effectiveness of NBSP in reducing loneliness is growing, there is a clear research gap regarding health economic evaluations of such interventions [17, 18]. In particular, there is a lack of cost-utility, cost-effectiveness, and cost-capability studies of NBSP interventions conducted alongside randomized controlled trials (RCTs) [19]. In times of restricted healthcare budgets, more evidence on resource utilization, costs, and cost effectiveness of new health interventions is necessary [20]. There is rising demand to assess cost-effectiveness alongside the trial next to the effectiveness of an intervention, to inform policy and decision makers about whether an intervention provides good value for the resources invested. Economic evaluations conducted alongside the trial are considered among best practice methods for systematic analysis of cost effectiveness [21]. RCTs can facilitate health economic evaluations by collecting data on the costs of the intervention and assessing the trade-offs between costs and effectiveness [22].

This study protocol is framed within the RECETAS (Re-imagining Environments for Connection and Engagement: Testing Actions for Social Prescribing in Natural Spaces) study [23]. The aim of this article is to present a site-specific study protocol for the health economic evaluations conducted alongside three RECETAS RCTs to evaluate whether NBSP is cost effective as a loneliness-reduction strategy.

## Methods

This methods section describes the framework elements and steps of our health economic evaluation, including: the RECETAS trial design, study setting and population, interventions and comparators, measurement and valuation of outcomes/ benefits, resource utilization and costs, statistical analysis, sample size and missing data, health economic evaluation designs of the cost-utility analysis (CUA), cost-effectiveness analysis (CEA), cost-capability analysis (CCA), and responder analysis, as well as sensitivity and scenario analyses.

### RECETAS trial design

RECETAS is an EU H2020 project (No 945095) that specifically addresses the social determinants of health and analyzes the impact of NBSP interventions on outcomes including loneliness, health-related quality of life and capability well-being, across three registered RCT study sites, and examines its qualitative aspects in three pre-post observational study sites [24]. The study protocol for the RECETAS trials describing the eligibility criteria and a logic model explaining the RECETAS project and the NBSP interventions has been published earlier [23, 24]. The RECETAS project addresses loneliness and aims to assess effectiveness, costs, and cost effectiveness of NBSP on loneliness in six different study sites across Europe, Latin America, and Australia [24]. Three pre-post observational studies in Cuenca (Ecuador), Marseille (France), and Melbourne (Australia) consider the relationship between NBSP and loneliness using qualitative methods [25]. Three randomized controlled trials (RCTs) are conducted in Barcelona (Spain), Prague (Czech Republic), and Helsinki (Finland). Three separate and site-specific health economic evaluations of NBSP for Barcelona, Prague, and Helsinki will be performed based on the data collected in the respective RCTs [23, 26]. The effectiveness and cost effectiveness of the two strategies (NBSP intervention vs. control group) within the RECETAS project will be assessed after three months at the end of a NBSP intervention, and at six-, and twelve-months post-randomization. The health economic evaluations alongside the trials will quantify the resources utilized in both arms and the additional costs and incremental health outcomes of NBSP compared to usual care from a societal and/or healthcare perspective and will include incremental cost-utility, cost-effectiveness, and cost-capability analyses.

International guidelines, such as the International Society for Health Economics and Outcomes Research (ISPOR) Good Research Practices Task Force Report on Cost-effectiveness Analysis alongside Clinical Trials II [27], Methods for Health Economic Evaluations - A Guideline Based on Current Practices in Europe [28], and the Consolidated Health Economic Evaluation Reporting Standards 2022 (CHEERS 2022) [29] will be followed for analyzing and reporting the results. Each intervention site obtained ethical approval by the respective Ethics and Research Committees (Barcelona: Research Ethics Committee at UVic-UCC, registration number 2014/2022 on 28 June 2022; Primary Health Care Research Institute of Catalonia, registration number 22/170-P on 02 August 2022; The Ethics Committee for Research with Medicines at IRIS-CC, registration number 23/016 on 11 October 2023; Prague: Ethics Committee of the Faculty of Humanities, Charles University on 28 June 2022; Helsinki: University Hospital Ethics Committee (No HUS/119/2022), Social Services and Health Care of the City of Helsinki on 13 June 2022) [23]. Ethical approval at UMIT TIROL – University for Health Sciences and Technology was also obtained from the Research Committee for Scientific Ethical Questions (registration number: 3274 on 19 June 2023).

### Study setting and population

The study population of our health economic evaluations alongside the trial depends on the three different study sites: In Barcelona, adults over 18 years living in socio-economically vulnerable areas suffering from loneliness are selected (RECETAS-BCN trial) [23, 25]. In Prague, older people (60+) with a risk of social isolation and loneliness are included (RECETAS-PRG trial). For the study carried out in Helsinki, older people (55+) living in local assisted living facilities (retirement homes), some with mild-moderate cognitive impairment are included (RECETAS-HLSNK trial) [25].

### Interventions and comparators

In all three RCTs (Barcelona, Prague, and Helsinki), participants are randomized to an intervention or control arm. Detailed descriptions of both arms have been published previously [23, 30]. Briefly, in the intervention arm, participants participate in the “Friends in Nature” (FIN) intervention, an adaptation from the Circle of Friends^®^ methodology developed by the University of Helsinki [30], “customized to the specific target population in each trial and with a focus on nature-based activities” [23]. Two trained facilitators are assigned to each group to support the group dynamics by fostering empowerment and, at the end of the group process, independence from the facilitator. The training and mentoring process of the facilitators based on the Circle of Friends^®^ methodology has also been described previously [23, 30]. In the first session, an individualized one-to-one interview with the facilitators is conducted in Barcelona and Prague. Individuals are then allocated to take part in a program of weekly nature-based group activities (5–12 people) for nine sessions [23]. The activities vary from week to week, and include a combination of strategies promoting empowerment, social connectiveness, and participation in nature-based activities. The group stays together, and they discuss and decide on the activities. During this time, the facilitators keep a diary, instruct the group, assure connectivity, and assist in ensuring compliance. The control group receives usual care (UC). In Barcelona and Prague, UC consist of an individual NBSP interview at which participants receive a menu of nearby nature-based community resources to utilize individually, without further support [23]. Potential uptake of nature-based activities following the interview will not be considered in our health economic evaluations. In Helsinki, control participants receive normal care, which includes socializing in their ward, occasional recreational activities and some outdoor activities. Importantly, they do not participate in any further organized activities and receive no support from the FIN group or the facilitators.

### Measurement and valuation of outcomes/ benefits

For the health economic evaluation in Barcelona and Prague, the effectiveness of the intervention will be measured by comparing health-related quality of life (HRQoL), loneliness, and capability against the control group. Capabilities are applied to measure wellbeing beyond health [31]. In Helsinki, the effectiveness of the intervention will be assessed by comparing HRQoL and loneliness only. In Helsinki, by agreement with the responsible trial partners, capabilities will not be assessed as a large proportion of the participants may be cognitively unable to respond reliably and no Finnish translation of the respective measurement instrument (i.e., ICECAP-A) is available.

A cost-utility, cost-effectiveness, and in Barcelona and Prague, a cost-capability analysis will be performed. In addition, a responder analysis will be conducted [32, 33]. Results will be interpreted with priority given first to the cost-utility analysis, followed by the cost-effectiveness analysis, the cost-capability analysis, and finally the responder analysis.

Table 1 summarizes the measured health outcomes, instruments applied, time points, study sites, where the outcome will be measured, and data sources.

**Table 1:** Overview of measured health outcomes

Health Outcome	Instrument	Time Points	Study Sites	Data Source
Health-related quality of life	EQ-5D-5L	t0, t1, t2, t3	Barcelona, Prague, Helsinki	Trial (Self-reported outcomes, proxy version by facilitators/ study nurses)
Loneliness	De Jong Gierveld Loneliness Scale	t0, t1, t2, t3	Barcelona, Prague, Helsinki	Trial (Self-reported outcomes)
Capability	ICECAP-A	t0, t1, t2, t3	Barcelona, Prague	Trial (Self-reported outcomes)

*EQ-5D-5L* European Quality of Life 5 Dimensions 5 Level, *ICECAP-A* ICEpop CAPability measure for Adults t0: baseline, t1: after 3 months; t2: after 6 months; t3: after 12 months

For the primary cost-utility analysis (CUA), HRQoL will be measured by the generic quality-of-life instrument EQ-5D-5L [34, 35], which will be translated into utilities using national tariffs where available. The EQ-5D-5L is an established instrument to assess HRQoL and recommended to be used in health economic evaluations [36, 37]. For countries without an EQ-5D-5L tariff but with a value set for the three-level version of the EQ-5D, the mapping methodology will be applied [38]. Where no value set exists for the EQ-5D-5L in the country concerned, the most appropriate European value set will be chosen [38]. Table 2 gives an overview of the applied valuation approaches for HRQoL For Spain, the EQ-5D-5L is a valid instrument to measure perceived health [39] and a country-specific value set is available to calculate health utility scores [40]. For the Czech Republic, no value set exists so far and national guidelines recommend to use the UK tariff [41, 42] as done in a previous study [43]. However, there is no empirical justification for designating a particular tariff as the default and it is advised to apply a value set from a country that is comparable [38]. A recent study by Laszewska et al., 2022 developed supra-national value sets based on five criteria “cultural differences, religion, language, healthcare system typology, healthcare system financing and sociodemographic aspects” [44]. The Czech Republic was identified to have strong links to Poland, for which a EQ-5D-5L value set exists [45], within these five criteria [44]. For Finland, only a value set for the EQ-5D-3L exists [46], which can be mapped to the five-level version using the crosswalk approach [47, 48]. The study by Laszewska et al., 2022 grouped Finland within the Nordic cluster of countries, for which the Danish value set was the only one available [44]. In the absence of a national value set, the Danish EQ-5D-5L value set, next to the UK value set, has been commonly used as a proxy in Finnish economic evaluation studies. If, during the duration of the study, a more relevant value set for one of the participating countries becomes available, this will be used.

**Table 2:** Overview of applied valuation approaches for health-related quality of life

Study Site	Instrument for HRQoL	Value Set	Source
Barcelona, Spain	EQ-5D-5L	Spain	Ramos-Goñi, Craig [40]
Prague, Czech Republic	EQ-5D-5L	Poland	Golicki, Jakubczyk [45]
Helsinki, Finland	EQ-5D-5L	Denmark	Jensen, Sorensen [49]

*EQ-5D-5L* European Quality of Life 5 Dimensions 5 Level, *HRQoL* Health-related quality of life

The EQ-5D-5L questionnaire assesses five dimensions mobility, self-care, usual activities, pain/ discomfort, and anxiety/ depression with respectively five levels to be indicated by the respondents (no problems, slight problems, moderate problems, severe problems, and extreme problems) resulting in 3,125 health states [50]. A health-state utility value (HSUV) is attached to each of these specific health states. Utilities are a preference-based single number index ranging between 0 (dead) and 1 (perfect health). Quality-adjusted life years (QALYs) are derived by weighting the time in a health state by the HSUV, that is multiplying the HSUV with the time each individual is in the health state [50] and calculating the area under the empirical utility curve for the exact time until the final follow-up after twelve months or death. The EQ-5D-5L questionnaire will be distributed to and filled out by the participants in Barcelona and Prague. However, in Helsinki, the EQ-5D-5L questionnaire will be used as a proxy version filled out by facilitators or study nurses. The proxy questionnaire is provided for participants who are unable to answer the original questionnaire due to cognitive impairments.

Loneliness will be measured with the validated 11-item De Jong Gierveld Loneliness Scale (DJGLS), which offers three or five response possibilities [51, 52]. The overall score ranges from 0 to 11 and categorizes loneliness into three stages: 0–2: not lonely, 3–8: moderately lonely, 9–11: strongly lonely [52]. Loneliness will be assessed in different time intervals between baseline (t0), at the end of the NBSP intervention after three months (t1), at the end of a maintenance phase after six months (t2), and at the end of the follow-up period of the project after twelve months (t3). Loneliness-free days (LFD) will be calculated by transforming the DJGLS score from 0 to 11 to a loneliness value between 0 and 1. A score of 0–2 (meaning that an individual feels not lonely) on the DJGLS will be assigned with a value of 1 (having a “Loneliness-free day”), whereas a score of 9–11 (meaning that an individual feels strongly lonely) will be assigned with a value of 0 (having a “Loneliness day”). Days with intermediate scores of 3 to 8 (meaning that an individual feels moderately lonely) will be assigned a value between 1 (“Loneliness-free day”) and 0 (“Loneliness day”) by linear interpolation. This approach is in line with the approach used by a previous study using depression-free days as health outcome [53]. The number of LFD over time will then be calculated by multiplying the loneliness value (from 0 to 1) with the days (e.g., 90, 180) in this loneliness state [54]. A hypothetical example with a graphical representation of the LFD calculation can be found in the Appendix A1. The change in loneliness is used as the measure for effectiveness in the cost-effectiveness analysis (CEA). Also in the responder analysis, a responder will be defined as a participant achieving a reduction of two points or more on the DJGLS at least once within the 12-month follow-up period.

As the EQ-5D-5L questionnaire is used to value a person’s health-related quality of life but might not capture all aspects of social well-being, we will perform an additional cost-capability analysis (CCA). Therefore, the ICECAP-A questionnaire will be used. The ICECAP-A questionnaire comprises additional aspects of well-being including dimensions beyond mere health and such as attachment, stability, achievement, enjoyment, and autonomy [31, 55]. These five dimensions are assessed on four levels resulting in a total of 1,024 capability states. For ICECAP-A, different value set exists [56–59]. We will use the original UK tariff as referred to by the University of Bristol [60]. After scoring a value for each of the five dimensions, the value for the overall capability state is calculated by summing them up. A capability state can have a value from 0 (no capability) to 1 (full capability). By multiplying capability values with the dwelling time in the capability state, years of full capability (YFC) are calculated [57]. The approach for calculating YFC is similar to calculating QALYs, however capability states are used instead of health states. The ICECAP-A questionnaire will be included in the trials in Barcelona and Prague.

Data from the EQ-5D-5 L [61], the DJGLS [52], and the ICECAP-A questionnaire [31] are collected at t0, t1, t2, and t3.

### Resource utilization and costs

The assessment of resource utilization and direct and indirect cost elements are included in the economic evaluation. Table 3 gives an overview of the different direct and indirect healthcare and non-healthcare cost items and includes information on the data sources for the valuation of utilization, unit prices and the corresponding perspective. The quantity of resources consumed by the participants and for the organization during the intervention will be derived from each trial site separately. Data on the use of health and social services during the entire study period will be collected using a RECETAS health economic questionnaire, registries, electronic medical records, and published literature. All items of the RECETAS health economic questionnaire are based on self-reported data or reported from the study nurse from the participants at t0, t1, t2, and t3. The RECETAS health economic questionnaire was developed based on published and unpublished literature [62–68]. Additionally, the questionnaire was discussed and adapted with each study site to ensure alignment with country-specific contexts and reflect the national healthcare setting, but it was not formally validated by participants or piloting. The questionnaire was part of the training of staff administering the questionnaires and is available from the authors upon request.

**Table 3:** Overview of costs

Type of Cost	Resource and Cost Items	Specification/ Examples	Time Points	Perspective	Source of Resource Utilization	Potential Source of Unit Price
Direct Non-healthcare Costs	NBSP Preparation and Implementation Costs	Menu development, training facilitators, advertisement, insurance	t0, t1	Healthcare, Societal	Trial	Trial
	NBSP Intervention Trial Costs	Transportation, entrance fees, facilitators salary	t0, t1	Healthcare, Societal	Trial	Trial
Direct Healthcare Costs	Outpatient Hospital Services	General practitioner, specialist (e.g., psychiatrist, cardiologist), emergency care, nurse	t0, t1, t2, t3	Healthcare, Societal	Health economic questionnaire (self-reported), medical records	National physician fee schedules/ health services tariffs
	Inpatient Hospital Services	Emergency care physician (hospital), psychiatric center, general medical ward, general surgical ward, rehab clinic	t0, t1, t2, t3	Healthcare, Societal	Health economic questionnaire (self-reported), medical records	Hospital billing system, national Diagnosis Related Groups
	Health Care Professional (Home Visits)	General practitioner, nurse, physiotherapist	t0, t1, t2, t3	Healthcare, Societal	Health economic questionnaire (self-reported), medical records	Reimbursement rates from national physician fee schedules
	Primary Social Care	Home care worker, social worker, ambulance transport, community/ social services	t0, t1, t2, t3	Healthcare, Societal	Health economic questionnaire (self-reported), medical records	Health services tariffs
	Medication	Prescribed medication, non-prescribed medication	t0, t1, t2, t3	Healthcare, Societal	Health economic questionnaire (self-reported), medical records	National drug price database
Indirect Non-health care Costs	Time Costs	Informal/ Community care (e.g., relatives, neighbors), Participants	t0, t1, t2, t3	Societal	Trial	Average salary or minimum wage according to National Statistics
	Productivity Costs	Absenteeism (labor market earnings lost)	t0, t1, t2, t3	Societal	Trial	Average salary or minimum wage according to National Statistics

t0: baseline; t1: after 3 months; t2: after 6 months; t3: after 12 months

Direct costs related to the resource utilization of health care, such as medication, hospital stays, and physician visits, as well as the non-healthcare costs of the NBSP intervention will be included [69]. The NBSP preparation and implementation costs include, amongst others, the training and working time of the facilitators, the development of the NBSP menu and the advertisement and communication as part of the organization of the trial. Insurances specifically relating to the delivery of the NBSP intervention (e.g., insurance coverage in case of injury during group sessions or accidents occurring during activities in natural environments) will be included in the healthcare perspective as the insurance is paid by the healthcare payer and represents a direct cost of delivering the intervention. However, under the societal perspective, most of the insurance premiums are transfer costs in case of accident compensation with a negligible administrative cost ratio of typically less than 1%. Therefore, insurance premiums are not included in the societal perspective, as they represent mostly a redistribution of financial resources from the payer institution to the insurance provider [70, 71]. NBSP intervention trial costs include the transportation and the facilitators earnings, which will be calculated by multiplying hours worked by their average salary. The transportation costs of the facilitators and transportation to the intervention site will be considered. As the intervention primarily takes place in publicly accessible green or blue areas, although some nature-based activities are organized indoors, almost no venue costs are expected to arise. For the societal perspective, out-of-pocket costs for the usual care participants are collected regarding participation costs for comparable group activities in nature, such as equipment or transportation. Session-specific costs paid by the participants in the intervention group will also be considered (e.g., food and beverages).

The choice of the perspective of the economic evaluation is based on the study population and on national and methodological guidelines for economic evaluations of the trial sites [28, 72–74]. In Barcelona, the study population includes adults aged 18 years and older, and following the national recommendations a societal and a healthcare perspective is adopted [72]. For the societal perspective, indirect non-healthcare costs beyond the healthcare sector, such as time costs for community care and costs due to productivity losses will be assessed [69, 75]. The community care costs are based on informal care comprising the value of unpaid caregiver time or uncompensated household production. Costs due to productivity losses are related to the absenteeism of work or reduced productivity, such as labor market earnings lost or less earnings due to loneliness. The impact of valuing these productivity costs using the friction cost method will be explored [75, 76]. According to the friction cost approach, indirect costs due to productivity loss can be calculated by multiplying the incidence of unemployed participants with the minimum (or average) salary in Spain over the friction period, that is the average time to replace the employee (i.e., six weeks or end of the follow-up, whatever occurs earlier) [77, 78]. For the Czech Republic, the national guidelines recommend to adopt a healthcare perspective for economic evaluations [28, 73]. Although recommended generally in Finland [74], a societal perspective for costs will not be applied in the Helsinki trial because the target population are older people permanently residing in assisted living facilities and indirect costs referring to productivity losses and time costs are less relevant. Thus, the economic evaluation in Helsinki will be performed from a healthcare perspective.

In general, costs will be calculated by multiplying the amount of resources consumed by their corresponding country-specific prices. Costs of the NBSP intervention and control group will be accumulated over the time horizon of the trial and reported as total costs and as mean costs per participant in Euros (EUR) for Barcelona and Helsinki or Czech Koruna (CZK) for Prague. Costs for inpatient hospital services will be based on national Diagnosis Related Groups fees. Healthcare professionals and primary social care will be calculated by multiplying the number of contacts or hours of consultations with the national physician fee schedules or respective health services tariffs. Medication costs will be calculated by multiplying the dosage by the frequency and standard price over the one-year trial period. In addition, short-term temporary medications might be considered. Non-prescribed medications reflecting out-of-pocket payments for the participants will be considered only in the societal perspective. All costs will be adjusted to a standard reference year if necessary, using the consumer price index [79]. Due to the short time horizon of the trial, future costs and health outcomes are not discounted to present values [80, 81].

### Statistical analysis

As described in the study protocol for the RCTs, the main analysis will be carried out according to the intention-to-treat (ITT) principle, that is, all participants will be analyzed according to the arm they were randomly assigned to [23]. In alignment, our health economic evaluations will also follow the ITT approach.

Descriptive analysis and statistical tests for the comparison of differences in baseline variables between the groups will be performed. Depending on the type and distribution of the variables (e.g., resource utilization, HSUV, loneliness, capability), different statistical methods will be applied. Normally-distributed continuous variables will be compared by analyzing their means and standard deviations using parametric test procedures, such as the Students t-test [82, 83]. Non-normally distributed continuous variables will be analyzed using non-parametric tests, such as the Mann-Whitney U test, focusing on medians and interquartile ranges [82, 83]. Categorical variables will be compared using the chi-square test [84]. All tests will be two-sided with a significance level set at α = 0.05.

Health effects and costs will be reported as mean differences to be used in the cost-utility/ -effectiveness/ and -capability analysis [85]. To account for the typically skewed distribution of cost and outcome data, regression-based methods, primarily ordinary least squares, combined with non-parametric bootstrapping will be used to estimate mean differences and corresponding confidence intervals without relying on parametric assumptions [86–90]. If the data exhibit extreme skewness or if bootstrapping is deemed unsuitable, alternative methods will be explored. In line with current recommendations, generalized linear models may be applied as an alternative analytical strategy where appropriate [89]. In the presence of significant differences in baseline characteristics between the groups, such as in socio-demographic factors or utility values, adjustment will be made by using multivariable regression-based methods [27, 86].

In case of outliers in cost data, winsorizing will be applied in sensitivity analyses [91, 92]. Outliers will be classified using the interquartile range (IQR). Values lying outside a range of 1.5 times IQR above the 75th percentile or below the 25th percentile will be defined as outliers [91]. Outliers will be replaced with the largest or smallest value not considering the outliers [91]. In case of strong correlation between costs and health outcomes [93], seemingly unrelated regression models to jointly analyze costs and health outcomes will be applied in sensitivity analyses [94].

RStudio and SAS software (SAS Institute Inc) will be used for statistical analyses [95].

### Sample size and missing data

The sample size for the clinical trials was determined based on detecting a significant difference in the main clinical outcome, HRQoL, measured by the 15D instrument [27, 90, 96]. The calculated sample size for each RCT is 316 patients, accounting for a potential 25% drop-out rate. The recruitment of participants was completed at the Barcelona study site in May 2024 and in Helsinki in September 2023. In Prague, 95% of participants were recruited by September 2024. Successfully recruited participants were randomized to an intervention and control group. For details of the sample size calculation, we refer to the study protocol of the RCTs of the RECETAS project [23]. Our health economic analyses are not based on sample size calculations and may, therefore, be underpowered. To assess uncertainty, we will use bootstrap-based confidence intervals (CIs) and cost-utility/ -effectiveness/ -capability acceptability curves (CUAC, CEAC, CCAC). Missing data must be treated carefully in economic evaluations [97]. Appropriate imputation approaches will be used [98]. Incompleteness in baseline data, including but not limited to age, HSUVs, loneliness, or capability, will be solved with multiple imputation [98]. For missing values during follow-up, imputation methods depending on nature of the variable, the mechanism of missingness and the percentage of missing values will be used [99]. For instance, linear interpolation (e.g., for missing in-between utilities), single imputation methods, such as the last value carry forward method (e.g., medication dose or long-term medication) [98], or multiple imputation (MI) methods such as the imputation chained equations method (MICE) will be used for skewed data (e.g., resource utilization) [98]. In case of missing at random, MI or likelihood-based methods are recommended [98]. For data missing completely at random, MI or inverse probability weighting can be applied and sensitivity analysis should be applied to test the impact of data being missing not at random [76, 98]. In case of informative censoring due to loss-to-follow-up, inverse-probability of censoring weights will be applied adjusting for covariates to control for informative censoring [89, 100].

### Health economic evaluation: CUA, CEA, and CCA alongside the trial

A comprehensive health economic evaluation, assessing differences in costs and health outcomes between the compared strategies will be conducted. These analyses will be conducted separately for each RECETAS trial site, based on the original individual data from the participants collected in Barcelona, Prague, and Helsinki. A pooled analysis will not be performed as primary analysis due to country-specific differences and target population [27]. However, it may be performed as an additional exploratory analysis depending on the characteristics of included participants to assess heterogeneity aspects and inform further research. Depending on the outcome measure, three different types of health-economic evaluations will be conducted to compare the “NBSP” strategy against the “No NBSP” strategy. The results of the health economic evaluations, which are incremental ratios as explained in Table 4, will compare the incremental costs to the incremental health outcome measures. The primary evaluation will be a cost-utility analysis using health-related utilities derived from EQ-5D-5L as health outcome measure. The incremental cost-utility ratio (ICUR) will be reported as the cost per QALY gained and reflects the ratio between the difference in costs and the difference in QALYs $\:\mathrm{(ICUR}\;=\frac{\triangle \mathrm{Costs}}{\triangle \mathrm{QALYs}}\mathrm{)}$. The second health economic evaluation will be performed in the form of a cost-effectiveness analysis evaluating the cost effectiveness in natural units of the clinical endpoints of the study. The incremental cost-effectiveness ratio (ICER) is the ratio of the difference in costs between the two strategies to the difference in effectiveness of the two strategies. For the CEA, we will use the change in loneliness as measured in LFDs with the DJGLS as outcome of effectiveness. By dividing the incremental costs with the incremental change in effectiveness, the ICER will be reported as the cost per LFDs $\:\mathrm{(ICER}\;=\frac{\triangle \mathrm{Costs}}{\triangle \mathrm{LFD}}\mathrm{)}$. In addition, a responder analysis will be conducted [32, 33]. A responder will be defined as a participant achieving a reduction of two points or more on the DJGLS at least once within the 12-month follow-up period. Responder rates will be compared between the intervention and usual care groups, and incremental costs per additional responder (${\mathrm{Responder}\;\mathrm{rate}}\;=\frac{\triangle \mathrm{Costs}}{\triangle \mathrm{Responders}}$) will be estimated. Uncertainty will be assessed using non-parametric bootstrapping and presented as CEAC.

Lastly, a cost-capability analysis will be applied using the differences in years of full capability derived from the ICECAP-A questionnaire as effectiveness measure. Based on the principle for calculating the ICER and the ICUR, the incremental cost-capability ratio (ICCR) will be calculated to report the cost per YFC gained $\:\mathrm{(ICCR}\;=\frac{\triangle \mathrm{Costs}}{\triangle \mathrm{YFC}}\mathrm{)}$. The analytic time horizon of the analyses is twelve months, aligning with the time horizon of the RECETAS trials.

**Table 4:** Overview of calculated incremental cost-utility, cost-effectiveness, and cost-capability ratios

	Incremental Costs (in EUR or CZK)	Incremental Effectiveness	Incremental Ratio
ICUR (EUR or CZK per QALYs gained)	$\triangle \mathrm{Costs}\;=\;\mathrm{Mean}\;\mathrm{Costs}_{\mathrm{NBSP}}-\mathrm{mean}\;{\mathrm{Costs}}_{\mathrm{UC}}$	$\triangle \mathrm{QALYs}\;=\mathrm{Mean}\;\mathrm{QALYs}_{\mathrm{NBSP}}-\mathrm{mean}\;{\mathrm{QALYs}}_{\mathrm{UC}}$	$\:\mathrm{ICUR}\text{}\mathrm{=}\frac{\triangle \mathrm{Costs}}{\triangle \mathrm{QALYs}}$
ICER (EUR or CZK per loneliness reduced)	$\triangle \mathrm{Costs}\;=\;\mathrm{Mean}\;\mathrm{Costs}_{\mathrm{NBSP}}-\;\mathrm{mean}{\mathrm{Costs}}_{\mathrm{UC}}$	$\triangle \mathrm{LFD}=\mathrm{Mean}\;\mathrm{LFD}_{\mathrm{NBSP}}-\mathrm{mean}\;\mathrm{LFD}_{\mathrm{UC}}$	$\:\mathrm{ICUR=}\frac{\triangle \mathrm{Costs}}{\triangle \mathrm{LFD}}$
ICCR (EUR or CZK per YFC gained)	$\triangle \text{ Costs}\;=\mathrm{Mean}\;\mathrm{Costs}_{\mathrm{NBSP}}-\mathrm{mean}\;\mathrm{Costs}_{\mathrm{UC}}$	$\triangle \mathrm{YFC}=\mathrm{Mean}\;\mathrm{YFC}_{\mathrm{NBSP}}-\mathrm{mean}\;\mathrm{YFC}_{\mathrm{UC}}$	$\mathrm{ICUR=}\frac{\triangle \mathrm{Costs}}{\triangle \mathrm{YFC}}$

*CZK* Czech Koruna, *EUR* Euro, *ICCR* Incremental cost-capability ratio, *ICER* Incremental cost-effectiveness ratio, *ICUR* Incremental cost-utility ratio, *NBSP* Nature-based social prescribing, *QALYs* Quality-adjusted life years, *UC* Usual care, *YFC* Years of full capability

The results of the economic evaluations will be shown in cost-utility, cost-effectiveness, and cost-capability planes, consisting of four quadrants representing the difference of effectiveness between the strategies on the x-axis and the difference in costs on the y-axis [101]. Before the calculation of the incremental ratios (i.e., ICUR, ICER, ICCR), dominance will be evaluated. A more effective and less costly strategy is called dominant and should be implemented. A dominated strategy or technology provides less benefits at higher costs and should be discarded. No incremental ratios will be calculated for dominant or dominated strategies [102]. Results will be interpreted with priority given first to the ICUR, followed by the ICER, and, finally, the ICCR.

### Uncertainty analyses

It is recommended that economic evaluations alongside the trial address uncertainty around the cost effectiveness [27]. For this purpose, scenario and sensitivity analyses will be performed. Scenario analysis will be performed to assess the robustness of the cost-utility, cost-effectiveness, and cost-capability results. Alternative cost assumptions, including scenarios where fixed costs to set-up the program are excluded and analyses without imputation will be conducted. For sensitivity analyses, the nonparametric bootstrap method will be used with 10,000 replications on the individual-level RECETAS data set [103]. The results of the sensitivity analyses will be presented as a scatter plot and 95% confidence ellipses on the cost-utility, cost-effectiveness, and cost-capability planes. The 95% CIs for bootstrap simulations will be determined using the percentile method [104]. Uncertainty related to further parameterized assumptions will be assessed via probabilistic sensitivity analyses.

Decisions on cost effectiveness often depend on willingness-to-pay (WTP) thresholds. The incremental ratios can be compared to the relevant WTP threshold for an additional effectiveness unit determined by the responsible decision-making authority. The intervention, that is NBSP, can be identified to be cost effective if the incremental ratio is below the specific WTP. However, only few countries have an explicit WTP threshold. For example, Schwarzer et al. describe WTP thresholds in ten countries and identified only the United Kingdom and Thailand as having an explicit WTP threshold value [105, 106]. For the Spanish National Health System, estimates of such WTP thresholds range between 21,000 Euro and 24,000 Euro [107] or 25,000 Euro and 60,000 Euro [108] per QALY gained for being cost effective. In Czech Republic, a WTP threshold of 1.2 million CZK/QALY (approx. 47,000 Euro/QALY) is recommended by the State Institute for Drug Control [73]. Finnish national guidelines do not report a WTP threshold [74].

No established WTP threshold currently exists for the outcome of our CEA expressed as changes in loneliness. Therefore, results will be presented without applying a predefined threshold and interpreted in terms of relative cost-effectiveness and uncertainty (e.g., using cost-effectiveness acceptability curves).

No official WTP threshold is defined for the ICCR or the YFC. In the UK, the current discussed thresholds range from £33,500 (approx. 39,214 Euro) to £36,150 (approx. 42,316 Euro) per YFC gained [109]. However, for the ICCR there is no official predefined WTP threshold for Spain, Czech Republic, and Finland that can be used for a NBSP intervention. In order to show the probability of being cost-effective, CEAC will be presented to summarize the parameterized uncertainty regarding the cost effectiveness and to show graphically the probability of the NBSP intervention being cost effective by considering different thresholds [104].

## Discussion

The demand from policy and decision makers for precise and structured assessments of the cost effectiveness of new interventions in health and social care is rising. In this context, our study protocol serves as a base for the systematic measurement and valuation of the benefits and costs of NBSP interventions alongside three RCTs in different settings and therefore site-specific requirements. We present the design of health economic evaluations to assess whether nature-based social prescribing is a cost-effective strategy against loneliness and its health-related problems. Transparency for the economic evaluation alongside the RECETAS trial is provided by stating our step-by-step procedure and using national and international guidelines [27, 28, 72–74, 104].

It is hypothesized that a NBSP intervention “in vulnerable people suffering from loneliness is more effective than usual social and health care” [110] in improving their quality of life and loneliness [110]. If reductions in healthcare usage (e.g., visits to general practitioners, medications, inpatient hospital services, social or community care) due to NBSP partially compensate its delivery costs accompanied with improvements in patient-related outcomes (i.e., increased quality of life, reduced loneliness, increased capabilities), it may also be a cost-effective strategy to alleviate loneliness.

Although RCTs are the gold standard of clinical studies to test the effectiveness of a new intervention, there are some limitations of economic evaluation studies alongside the trial. For instance, formal power calculations explicitly for the health economic evaluations were not performed. To summarize the uncertainty in the cost-effectiveness results, we will use cost-utility/ -effectiveness/ -capability acceptability curves. Due to ethical concerns, both arms received some kind of intervention depending on study sites, and, therefore, the contrast between the two arms may underestimate the actual effect of NBSP compared to no other activity. Furthermore, we will not consider potential uptake of nature-based activities in the control group in our health economic evaluations. Therefore, the analysis of before-after intervention effects in observational studies will complement the evidence. We also assume that, in future routine practice, neither implemented NBSP intervention will last forever nor will its effects. However, it may be that some of the baseline costs of the NBSP intervention will be counterbalanced by improved (moderate) long-term effects and related cost savings occurring after the one-year trial follow-up time horizon [27, 111, 112]. Additionally, we developed a health economic questionnaire with country-specific adaptations. The results of the planned health economic evaluations from the three site-specific conducted RCTs can vary across the three study sites and are limited depending on the corresponding setting and target population of Barcelona, Prague, and Helsinki. No pooled health economic analysis will be performed. Thus, generalizability outside the RCTs’ settings cannot be assumed, and the well-planned and structured environment of pragmatic trials brings a rather high internal but low external validity of the results. Therefore, our future research plan in the RECETAS project includes a decision-analytic modeling study assessing the long-term consequences of loneliness and the long-term cost effectiveness of NBSP under different settings, scenarios and assumptions [111]. Despite trying to assess all relevant costs of the NBSP intervention within the scope of the trials, it is possible that some costs will have been overlooked or not reported by the participants. Another limiting factor is that missing values and dropouts from the trial could appear, for example, due to the high number of items asked, the time point of the intervention, or competing events or commitments. However, data and results from the trials will be considered transparent and reproducible based on the study protocol. We have provided a clear and systematic framework for the health economic evaluations of NBSP interventions within the RECETAS project. Our study protocol offers a comprehensive, systematic and transparent approach for conducting health economic evaluations of NBSP interventions targeting vulnerable individuals suffering from loneliness and includes comprehensive uncertainty analyses. As a special feature, we perform economic evaluations using three different approaches including a CUA, CEA, and CCA capturing different components of health and wellbeing outcomes. The outcomes of the health economic evaluations will provide healthcare decision makers with crucial insights, supporting their consideration of NBSP as a viable intervention to reduce loneliness. 

## Figures and Tables

**Fig. 1:**
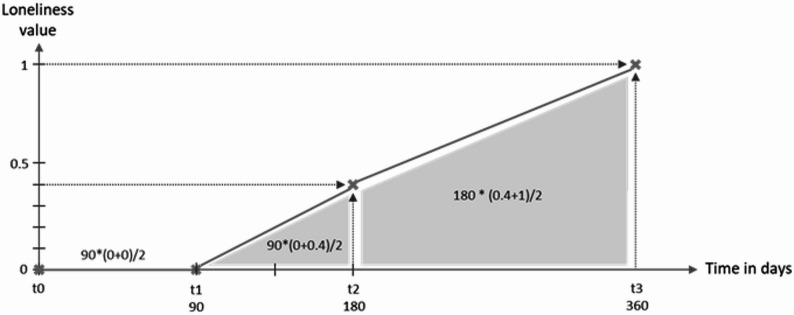
Calculation of loneliness-free days over four assessment time points (to, t1, t2, t3)

## Data Availability

No datasets were generated or analysed during the current study.

## Appendix

### A1. Calculation of loneliness-free days - hypothetical example

The following hypothetical example shows how loneliness-free days (LFD) will be calculated over four time points (t0, t1, t2, t3) assessing loneliness with the De Jong Gierveld Loneliness Scale (DJGLS) ranging from 0 to 11 (0-2: not lonely, 3-8: moderately lonely, 9-11: strongly lonely) (53). LFD will be calculated by transforming the DJGLS score to a loneliness value between 0 and 1. A score of 0-2 (not lonely) on the DJGLS will be assigned with a value of 1 (“Loneliness-free day”), whereas a score of 9-11 (strongly lonely) will be assigned with a value of 0 (“Loneliness day”). Days with intermediate scores of 3 to 8 (moderately lonely) will be assigned a value between 1 (“Loneliness-free day”) and 0 (“Loneliness day”) by linear interpolation. If an individual has a DJGLS score of 10 at baseline indicating strong loneliness, this is assigned with a loneliness value of 0. After three months, the person has a score of 9 on the DJGLS, what still indicates strong loneliness and is assigned with a loneliness value of 0. After six months, a score of 6 on the DJGLS indicates that the person is still lonely but moderately only what is assigned with a loneliness value of 0.4. And after twelve months, a score of 2 on the DJGLS indicating that the person is not lonely anymore, is assigned with a loneliness value of 1. Total LFD would be then calculated as follows: $\mathrm{LFD}=90\;\mathrm{days}*(0+0)/2+90\;\mathrm{days}*(0+0.4)/2+180\;\mathrm{days}*(0.4+1)/2=144$. Figure 1 visualizes total LFD by summing up the estimations for each interval.

## Abbreviations

CCA: Cost-capability analysis
CCAC: Cost-capability acceptability curve
CEA: Cost-effectiveness analysis
CEAC: Cost-effectiveness acceptability curve
CUA: Cost-utility analysis
CUAC: Cost-utility acceptability curve
CHEERS 2022: Consolidated Health Economic Evaluation Reporting Standards 2022
CI: Confidence interval
CZK: Czech Koruna
DJGLS: De Jong Gierveld Loneliness Scale
EQ-5D-5L: European Quality of Life 5 Dimensions 5 Level
EUR: Euro
FIN: Friends in Nature
HRQoL: Health-related quality of life
HSUV: Health-state utility value
ICECAP-A: ICEpop CAPability measure for Adults
ICER: Incremental cost-effectiveness ratio
ICCR: Incremental cost-capability ratio
ICUR: Incremental cost-utility ratio
ITT: Intention-to-treat
ISPOR: The Professional Society for Health Economics and Outcomes Research
ICR: Interquartile range
MI: Multiple imputation
MICE: Multiple imputation chained equations method
NBSP: Nature-based social prescribing
NHS: National Health Service
QALY: Quality-adjusted life year
RECETAS: Re-imagining Environments for Connection and Engagement: Testing Actions for Social Prescribing in Natural Spaces
RCT: Randomized controlled trial
SNM: Structural nested models
SP: Social prescribing
UC: Usual care
WTP: Willingness-to-pay
YFC: Years of full capability
